# Kidney biopsy findings in children with diabetes mellitus

**DOI:** 10.1007/s00467-023-06254-9

**Published:** 2023-12-21

**Authors:** Lasanthi Weerasooriya, Alexander J. Howie, Matthew P. Wakeman, Susan Cavanagh, David V. Milford

**Affiliations:** 1 Department of Nephrology, Birmingham Children’s Hospital, Birmingham, B4 6NH UK; 2 Department of Histopathology, Birmingham Children’s Hospital, Birmingham, B4 6NH UK

**Keywords:** Diabetes mellitus, Kidney biopsy, Diabetic nephropathy

## Abstract

**Background:**

Diabetic nephropathy may begin in childhood, but clinical kidney disease ascribable to this is uncommon in children with type 1 (insulin dependent) diabetes mellitus.

**Methods:**

We reviewed our experience of kidney biopsies in children with type 1 diabetes mellitus.

**Results:**

Between 1995 and 2022, there were biopsies in 17 children, with various clinical indications for kidney biopsy, making this the largest series of biopsies in diabetic children with clinical kidney abnormalities. Four biopsies showed diabetic nephropathy, three showed the combination of diabetic nephropathy and IgA nephropathy, and ten showed a variety of conditions other than diabetic nephropathy: minimal change disease (2), membranous nephropathy (2), thin glomerular basement membrane lesion (2), non-glomerular chronic damage in Wolcott–Rallison syndrome (2), acute pauciimmune necrotizing crescentic glomerulonephritis (1) and IgA nephropathy (1). Clinical clues of something other than diabetic nephropathy included acute kidney injury, microscopic haematuria or chronic kidney impairment with little or no proteinuria and the nephrotic syndrome after a short duration of diabetes.

**Conclusions:**

We confirm that changes better known in adults with either type 1 or type 2 diabetes mellitus can occur in children with type 1 diabetes mellitus: overt diabetic nephropathy either on its own or combined with other conditions and kidney disorders other than diabetic nephropathy.

**Graphical abstract:**

**Figure:**
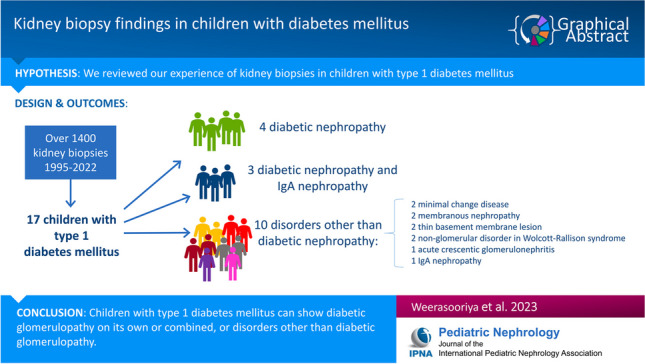
A higher resolution version of the Graphical abstract is available as Supplementary information

**Supplementary Information:**

The online version contains supplementary material available at 10.1007/s00467-023-06254-9.

## Introduction

Diabetes mellitus is a major health problem. Both type 1 (insulin dependent) and type 2 (insulin resistant) diabetes mellitus increased in incidence worldwide between 1990 and 2017, although type 1, most commonly beginning in childhood, was much less prevalent than type 2 [1]. In the UK in 2021, diabetes mellitus was the commonest identifiable kidney disease in adults aged at least 18 years old starting replacement therapy for kidney failure, although no children were reported to have diabetes mellitus as the reason to start replacement therapy [2]. This emphasises the length of time that diabetic nephropathy, the most likely explanation of kidney failure in diabetic people, takes to cause failure.

Most adults with type 1 diabetes who had a kidney biopsy were found to have diabetic nephropathy, while many with type 2 diabetes had a range of kidney disorders either instead of or as well as diabetic nephropathy, rather than just diabetic nephropathy [3]. Kidney biopsy is rare in diabetic children, although biopsy has been recommended to be used more often to detect the early stages of diabetic nephropathy, which ideally requires sophisticated morphometry on electron microscopic sections [4]. Few series have been published of kidney biopsies in diabetic children, compared with those in adults [5].

We reviewed our experience of kidney biopsies in diabetic children in a period of over 25 years.

## Materials and methods

Children were all under 18 years of age when seen at Birmingham Children’s Hospital. Records from 1995 onwards in the Departments of Nephrology and Histopathology were reviewed to find children who were known to have diabetes mellitus and had a kidney biopsy. Clinical records and all available material on kidney biopsies, including light microscopic and immunohistological sections and electron micrographs, were reviewed.

**Fig. 1:**
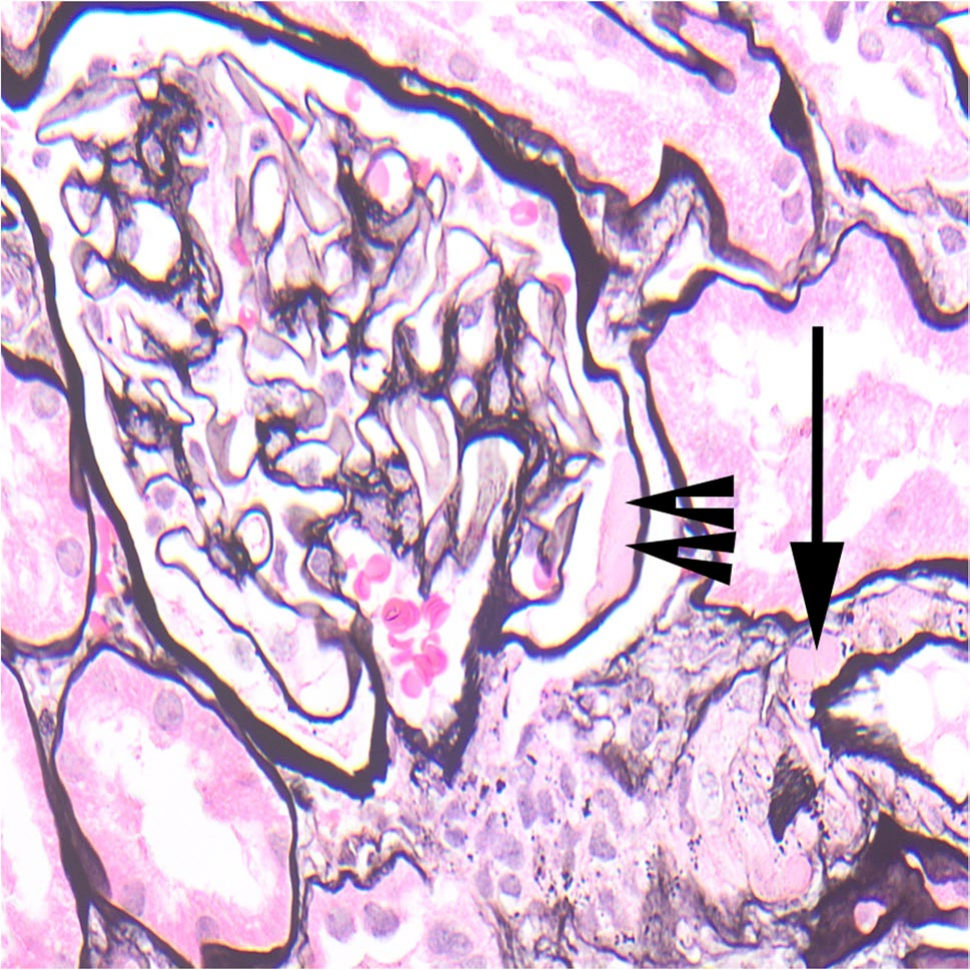
Glomerulus in a child with diabetic nephropathy, class 2a (case 4). There is mild mesangial expansion, with hyaline arteriolosclerosis (arrow) and a capsular drop (double arrowheads)

**Fig. 2:**
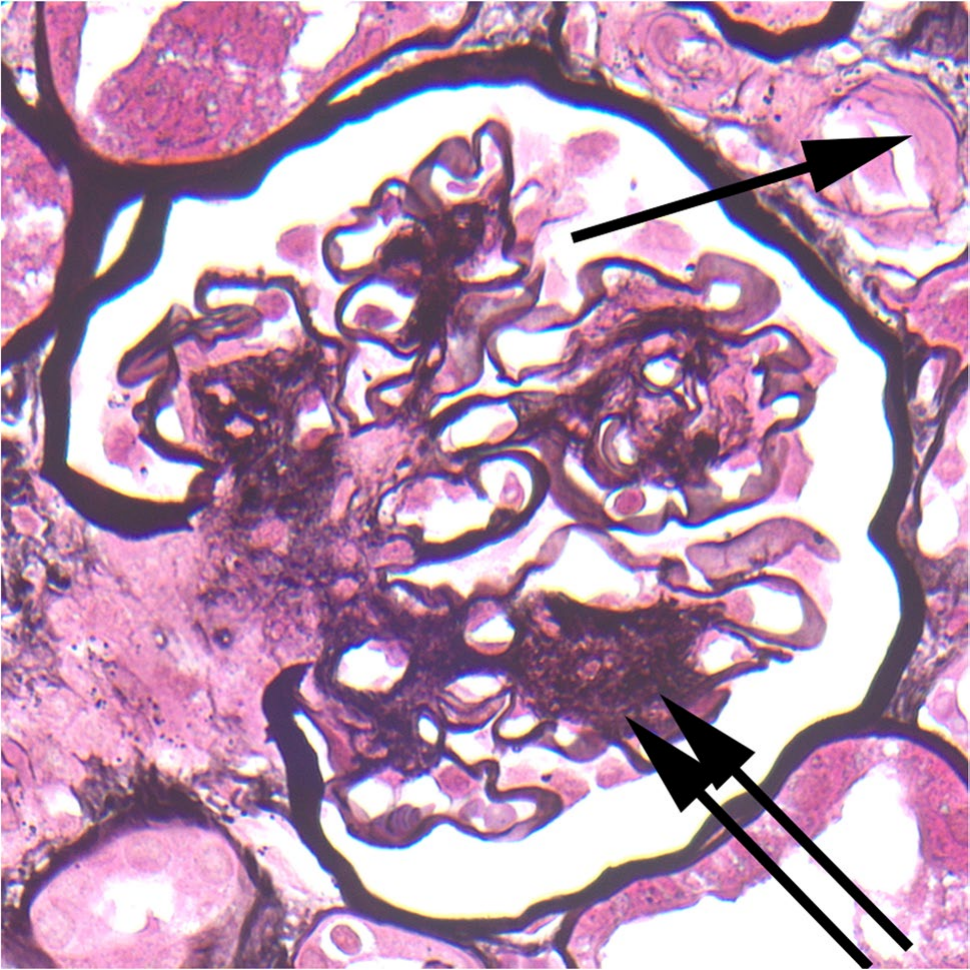
Glomerulus in a child with diabetic nephropathy, class 3 (case 3). As well as hyaline arteriolosclerosis (arrow), there is a mesangial nodule (double arrow)

Pathological diagnoses were made in standard ways [6]. In particular, diabetic nephropathy was identified by increased size of mesangium on light microscopy and increased width of glomerular basement membranes on electron microscopy, and often by various other features such as hyalinosis in arterioles, formation of capsular drops and mesangial nodules (Figs. 1 and 2) [5]. The Renal Pathology Society classification of diabetic nephropathy was applied: class 1, thickening of glomerular basement membranes only; class 2, mesangial expansion, mild (2a), or severe (2b); class 3, mesangial nodules; and class 4, advanced diabetic glomerulosclerosis [7], although no examples of either class 1 or class 4 were found in our series. In biopsies showing IgA nephropathy, in view of the limited reproducibility of subjective scoring of the Oxford MESTC classification of IgA nephropathy, and also of its inconsistent prognostic value, no MESTC scoring was done [8, 9].

The amount of chronic damage in kidney biopsies was measured using a morphometric method on light microscopic sections stained with periodic acid-methenamine silver [10]. Images of the cortex in a biopsy were captured on a computer, and an interactive image analysis system was used to outline areas of chronic damage, meaning areas with globally sclerosed glomeruli, atrophic tubules and interstitial fibrosis. These areas were measured in arbitrary units, and their proportion of the total cortical area in the biopsy was calculated as a percentage. This was called the index of chronic damage, which theoretically could range from 0 to 100%, and has prognostic value [10]. The index is 0% in a normal child.

**Fig. 3:**
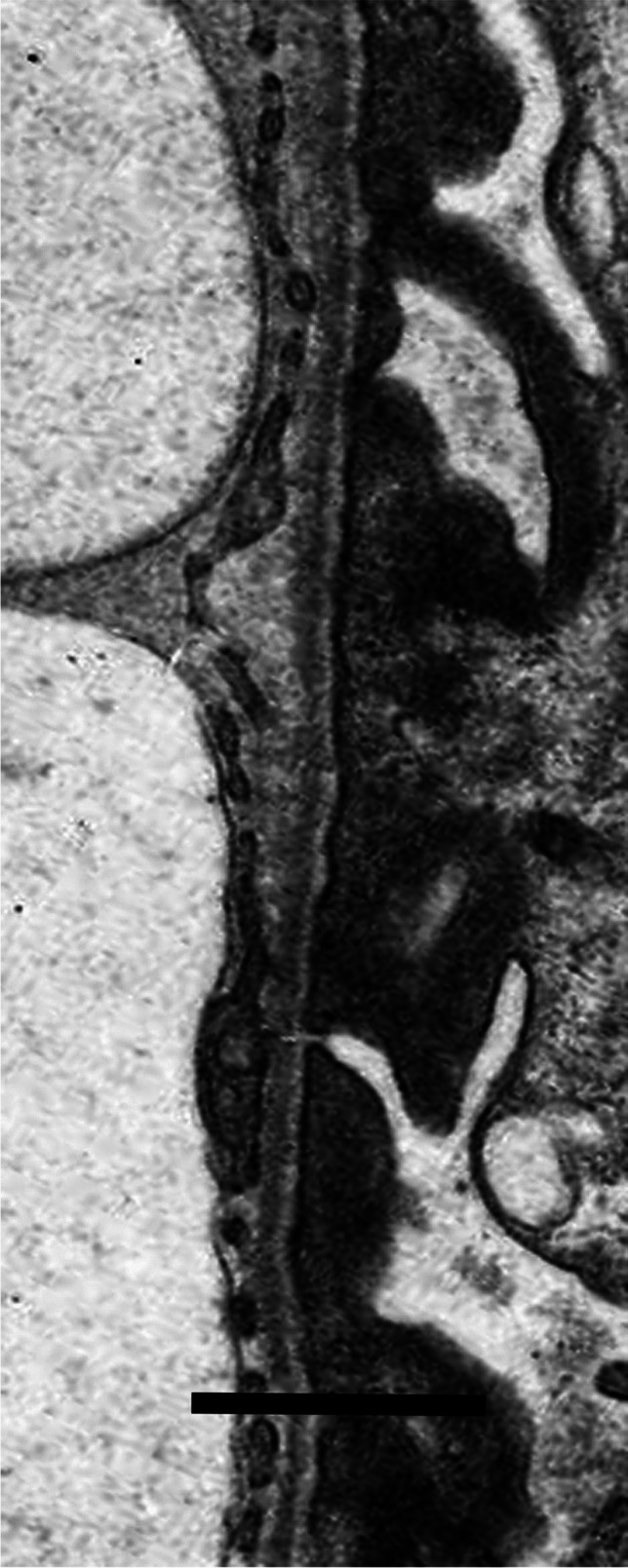
Electron micrograph of the glomerular basement membrane in a child with thin glomerular basement membrane lesion (case 13). Scale bar = 1 mm

**Fig. 4:**
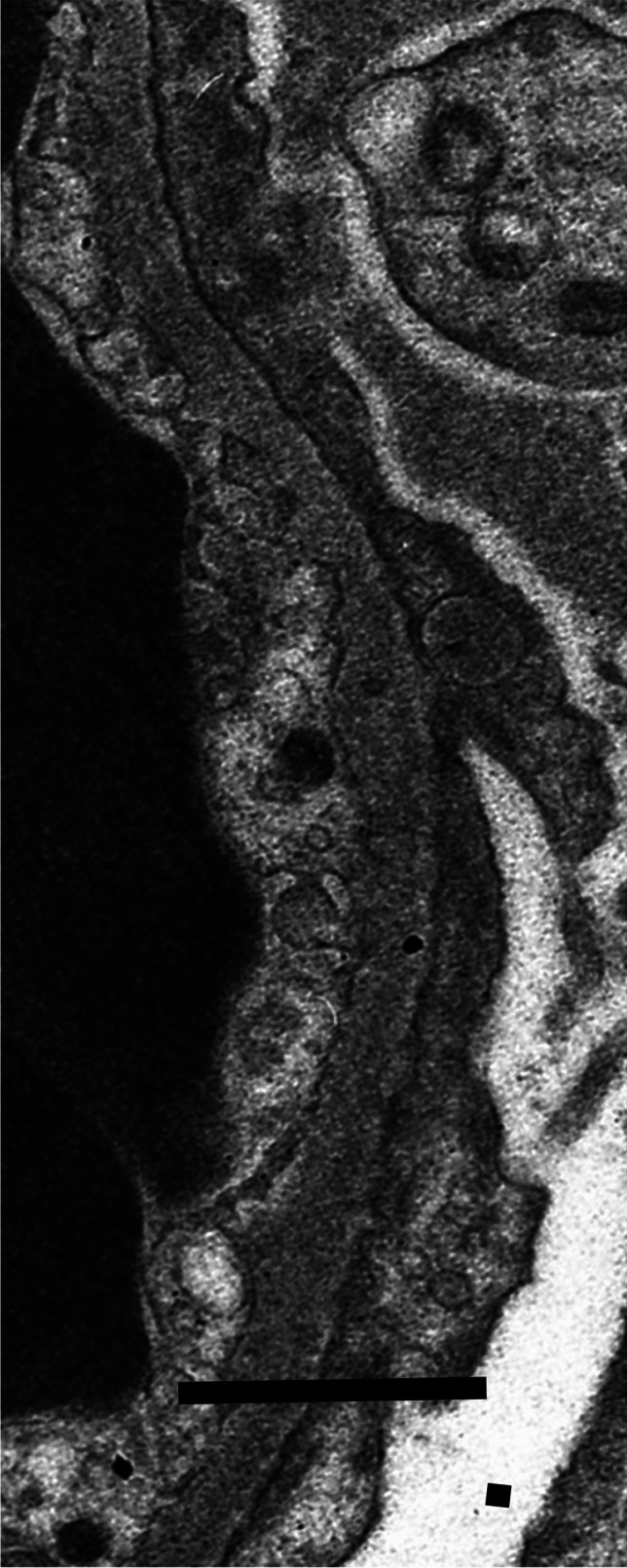
Electron micrograph of the glomerular basement membrane of normal thickness in a child with minimal change disease (case 10(1)). There is effacement of podocyte foot processes. Scale bar = 1 mm

**Fig. 5:**
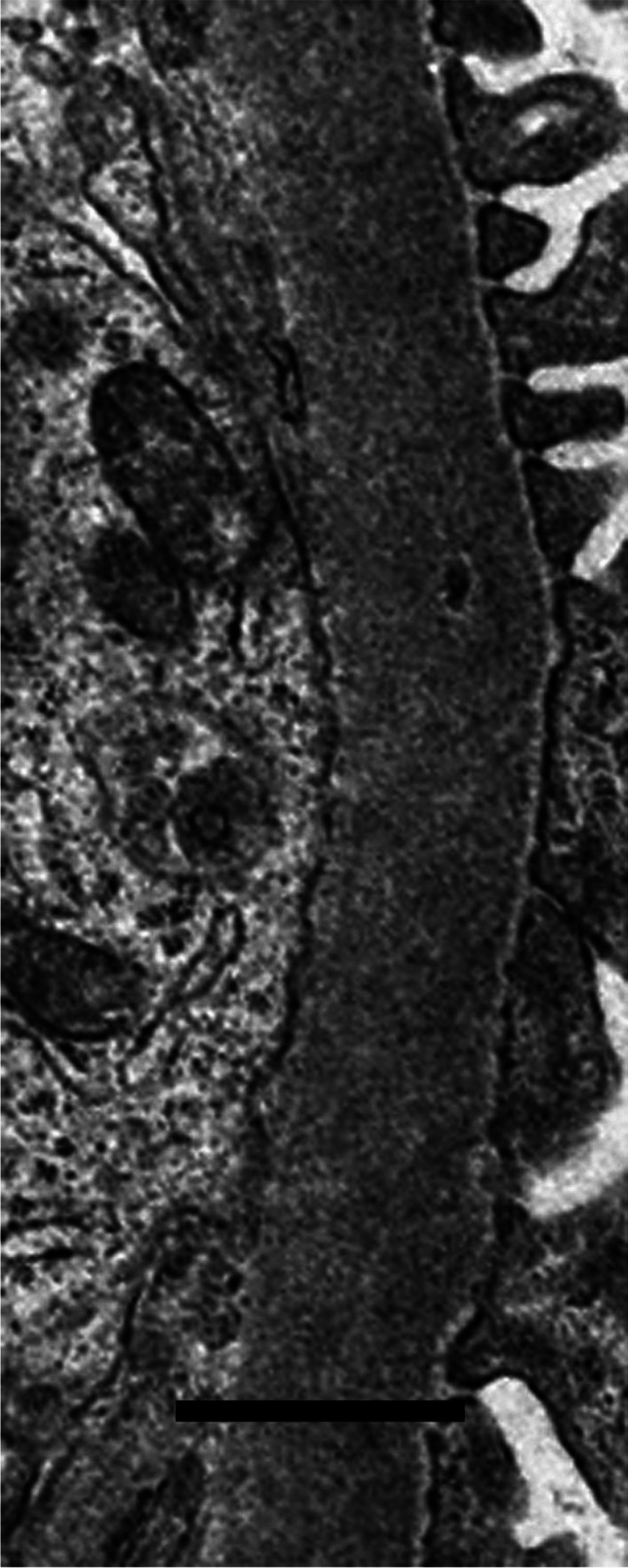
Electron micrograph of thickened glomerular basement membrane in the child with diabetic nephropathy illustrated in Fig. 1 (case 4). Scale bar = 1 mm

Available electron microscopic material, fixed and processed in various ways and photographed on different microscopes over many years, was not suitable for detailed retrospective morphometry of the thickness distribution of glomerular basement membranes, which required standardised fixation of specimens and preparation of electron micrographs with appropriate and accurate calibration of magnification; allowance for the age of a child using contemporary control biopsies; unbiased, systematic and independent sampling; an adequate number of measurements of orthogonal intercepts; and appropriate mathematical calculations [11, 12]. Instead, the pragmatic, subjective approach used by Haas [13] was followed, to identify not only abnormally thin glomerular basement membranes, as Haas did, but also with the necessary changes to identify abnormally thick basement membranes: “Most experienced renal pathologists are able to discriminate between glomerular basement membranes of normal thickness and those that are clearly thin, so in most cases it is not necessary to perform measurements” [13]. Figures 3, 4 and 5 show electron micrographs of glomerular basement membranes that are thin, normal and thick. In cases of doubt, the basement membranes were considered of normal thickness.

Counts were made of numbers of kidney biopsies at Birmingham Children’s Hospital and of children newly diagnosed with diabetes mellitus from 1995 to 2022 inclusive. Records were incomplete for some years, and estimated counts were made by extrapolation from averages of known yearly numbers.

## Results

Relevant kidney biopsies were identified from 17 children known to have type 1 diabetes mellitus, defined as requirement for treatment with insulin. One of these children had two biopsies. From 1995 to 2022 inclusive, there were approximately 860 children newly diagnosed with type 1 diabetes, and approximately 1440 native (nontransplant) kidney biopsies. In this time, under 2% of diabetic children had a kidney biopsy, and under 1.5% of kidney biopsies were included in this series.

Clinical features at the time of biopsy are shown in Table 1, plus pathological diagnoses, the index of chronic damage in biopsies and clinical outcomes. The diagnoses can be considered in three groups, as in Table 1.

**Table 1:** Pathological findings in kidney biopsies from 17 diabetic children, with clinical findings at biopsy and follow-up: numbers 1 to 4, diabetic nephropathy; 5 to 7, diabetic nephropathy plus IgA nephropathy; 8 to 17, kidney disorders other than diabetic nephropathy, arranged by age at initial biopsy within groups

Number in series; sex	Age at biopsy	Duration of diabetes at biopsy	Indication for biopsy	Other features at biopsy	eGFR	salb	UPr/cr	Pathological diagnosis	Index %	Age at follow-up	Clinical status at follow-up
1 F	12.5 y	73 m	pu	Diabetic retinopathy	123	40	656	Diabetic nephropathy class 2a	4	15.8y	CKD stage 1 eGFR > 95, UPr/cr 873
2 M	13.9 y	124 m	cki, pu	Down syndrome	62	31	294	Diabetic nephropathy class 2b	4	15.1y	CKD stage 3A eGFR 50
3 F	14.9 y	179 m neonatal onset	pu		nk	nk	nk	Diabetic nephropathy class 3	5	15.9y	CKD stage nk, UPr/cr 399
4 F	15.3 y	110 m	pu	FH DM	89	36	238	Diabetic nephropathy class 2a	0	16.4y	CKD stage 2 eGFR 88, UPr/cr 83
5 F	10.1 y	36 m	ns, mic ht	Cystic fibrosis	98	24	774	Diabetic nephropathy class 2b plus IgA nephropathy	0	17.5y	CKD stage 1 eGFR 108, UPr/cr 128
6 M	11.3 y	108 m	ns, cki, mac ht	HSP	34	35	166	Diabetic nephropathy class 3 plus IgA nephropathy	0	16.3y	CKD stage 3A eGFR 51, UPr/cr 20
7 M	13.3 y	79 m	cki, pu		73	41	76	Diabetic nephropathy class 2a plus IgA nephropathy	8	16.4y	CKD stage 2 cr 57, UPr/cr 76.6
8 F	5.9 y	19 m	ns		88	20	nk	Membranous nephropathy	2	17.5y	CKD stage 2 eGFR 68, UPr/cr 41
9 F	7.5 y	72 m	aki	ANCA positive (aMPO)	4	25	2755	Crescentic glomerulo-nephritis	68	10.5y	Dialysis then kidney transplant at age 10.5 y
10(1) M first biopsy	8.5 y	60 m	ns		87	21	3759	Minimal change disease	0	16.1y	See following
10(2) second biopsy	13.7 y	122 m	Cya treatment		nk	nk	nk	Slight CNI damage	1	16.1y	CKD stage 2 eGFR 74, still relapsing
11 F	8.7 y	104 m neonatal onset	cki	Wolcott–Rallison syndrome: small kidneys	62	39	< 20	Non-glomerular disorder	3	9.5y	CKD stage 3A eGFR 53, UPr/cr < 20
12 F	11.0 y	109 m	mic ht	FH DM	140	nk	nk	Thin GBM lesion	0	12.3y	CKD stage 1 “normal U&Es”, UPr/cr 11.7
13 F	12.2 y	72 m	mic ht	FH mic ht: father, mother, brother	88	42	< 2 0	Thin GBM lesion	1	12.4y	CKD stage 1, dipstick 2 + ht, trace pu
14 F	13.0 y	156 m neonatal onset	cki	Wolcott–Rallison syndrome: small kidneys	43	38	21	Non-glomerular disorder	3	18.4y	CKD stage 3B, UPr/cr 20
15 M	13.7 y	108 m	pu	aPLA2R, aTHSD7A negative	107	40	26	Membranous nephropathy	0	18.6y	CKD stage 2 eGFR 80, UPr/cr 21
16 F	14.9 y	36 m	cki, pu		53	35	91	IgA nephropathy	15	15.8y	CKD stage 3B cr 129, UPr/cr < 20
17 F	15.2 y	48 m	ns		101	15	423	Minimal change disease	0	16.2y	No CKD eGFR > 95, UPr/cr < 20

*aki* acute kidney injury, *aMPO* antibodies to myeloperoxidase, *ANCA* anti-neutrophil cytoplasmic antibodies, *aPLA2R* antibodies to phospholipase A2 receptor, *aTHSD7A* antibodies to thrombospondin type 1 domain-containing 7A, *CKD* chronic kidney disease, *CNI* calcineurin inhibitor, *cki* chronic kidney impairment, meaning reduced eGFR, *crescentic glomerulonephritis* pauciimmune necrotizing crescentic glomerulonephritis, *cya* ciclosporin, *DM* diabetes mellitus, *eGFR* estimated glomerular filtration rate, *F* female, *FH* family history, *GBM* glomerular basement membrane, *HSP* Henoch Schoenlein purpura, *ht* haematuria, *index* index of chronic damage, *M* male, *m* months, *mac ht* macroscopic haematuria, *mic ht* microscopic haematuria, *nk* not known, *ns* nephrotic syndrome, *pu* proteinuria, *salb* serum albumin concentration in g/L (normal range, 43–54 g/L), *U&Es* serum concentrations of urea and electrolytes, *UPr/cr* urinary protein/creatinine concentration ratio in mg/mmol (normal, under 20.0 mg/mmol), *y* years

### Diabetic nephropathy in diabetic children

**Fig. 6:**
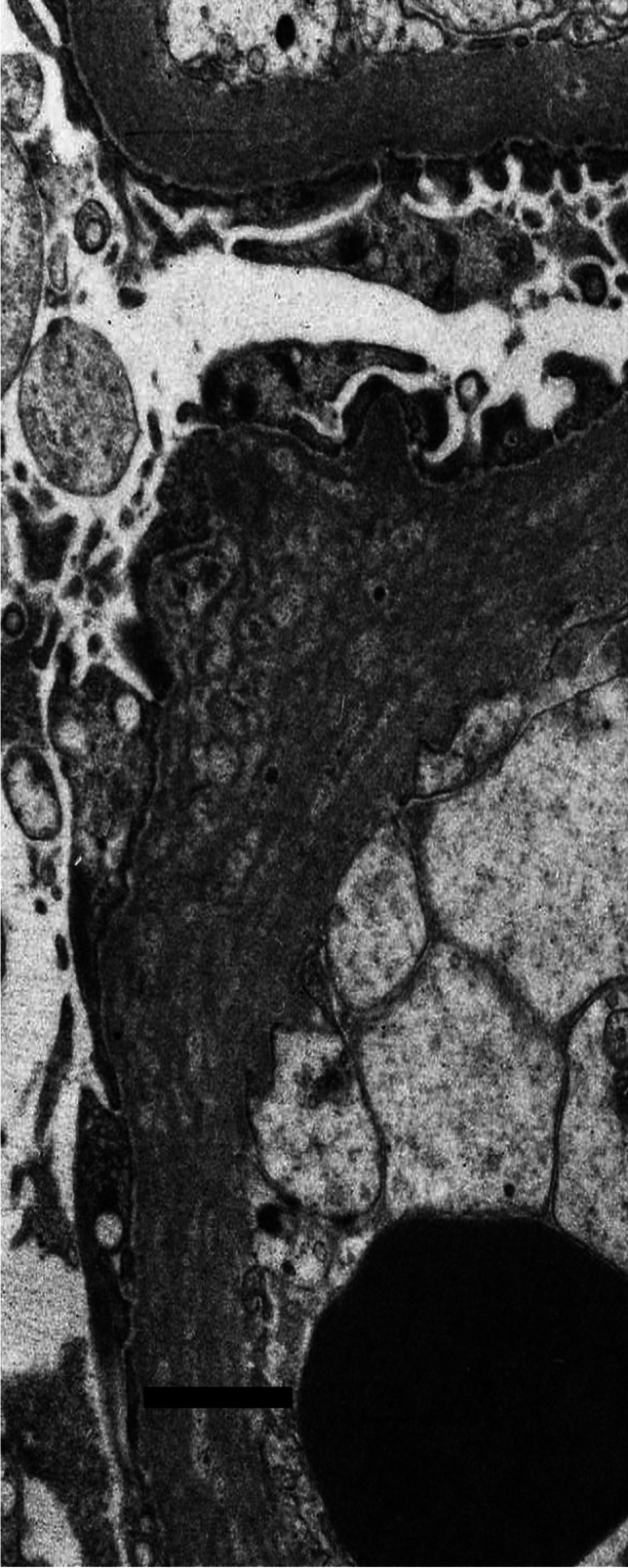
Electron micrograph of glomerular basement membranes in a child with diabetic nephropathy, class 2a (case 1), showing thickening that is irregular in places, with projections on the epithelial and endothelial aspects and a fibrillary appearance. Scale bar = 1 mm

Four children (cases 1 to 4) had changes of diabetic nephropathy without another glomerular disorder. One biopsy (case 1) also had vacuolation of the straight parts of proximal tubules (Armanni-Ebstein lesion). This was in the only child in the series with diabetic retinopathy. On electron microscopy, all biopsies showed thickening of glomerular basement membranes (Fig. 5), which in two was markedly variable, with irregular epithelial and endothelial surfaces in places and a fibrillary appearance in the membrane (Fig. 6) (cases 1 and 2).

Three children had a longer known duration of diabetes than the median of the series, 79 months. Three children were biopsied to investigate heavy proteinuria, which persisted at last follow-up. The other child was biopsied to investigate heavy proteinuria and kidney impairment, which also persisted. Three had an index of chronic damage more than the median of the whole series, 2%.

### Diabetic nephropathy plus IgA nephropathy in diabetic children

Three children had the combination of diabetic nephropathy and IgA nephropathy (cases 5 to 7). Each biopsy showed mesangial expansion on light microscopy with deposition of IgA on immunohistological study. Electron microscopy confirmed mesangial deposits and showed thickened glomerular basement membranes.

One child (case 5) had cystic fibrosis, with a homozygous delta F508 mutation in the cystic fibrosis transmembrane conductance regulator gene (*CFTR*). The biopsy was to investigate the nephrotic syndrome. Proteinuria persisted. Another child (case 6) had a history of Henoch Schoenlein purpura, and the biopsy was to investigate the nephrotic syndrome, with macroscopic haematuria and kidney impairment, which persisted. The biopsy in the third child (case 7) was to investigate proteinuria and kidney impairment, both of which persisted. This biopsy was the only one of the three with detectable chronic damage.

### Kidney disorders other than diabetic nephropathy in diabetic children

Ten children with no definite evidence of diabetic nephropathy on either light microscopy or electron microscopy had other disorders. Two children (cases 10 and 17) had minimal change disease (Fig. 4). In a later kidney biopsy, 5 years after the first biopsy and after treatment with ciclosporin, one (case 10) had evidence of mild chronic damage consistent with effects of a calcineurin inhibitor. Both children were initially biopsied to investigate the nephrotic syndrome, which responded to steroid treatment, with a relapsing course in one.

Two children had membranous nephropathy (cases 8 and 15). One (case 15), with mild proteinuria, had IgG4 in the glomerular subepithelial deposits, without evidence of antibodies to phospholipase A2 receptor (PLA2R) or thrombospondin type 1 domain-containing 7A (THSD7A). Later, this child developed neurological symptoms and was found to have serum IgG4 antibodies to contactin-associated protein-like 2 (CASPR2) and leucine-rich glioma inactivated 1 (LGI1) [14]. The other child (case 8), with the nephrotic syndrome, had no evidence of hepatitis B or C virus infection or systemic lupus erythematosus as the cause of membranous nephropathy, but no other relevant serological tests were available at the time.

Two unrelated children (cases 12 and 13) had glomerular basement membranes that on electron microscopy were undoubtedly thin for their age, and were diagnosed to have thin glomerular basement membrane lesion (Fig. 3). Both had persistent microscopic haematuria, with normal kidney function and slight or no proteinuria.

Two unrelated children with Wolcott–Rallison syndrome (cases 11 and 14) had a homozygous mutation in the eukaryotic translation initiation factor 2 alpha kinase 3 gene (*EIF2AK3*) [15]. They had neonatal diabetes mellitus, skeletal dysplasia, developmental delay and intermittent liver dysfunction. The kidney biopsies were to investigate kidney impairment without significant proteinuria. Both biopsies had evidence of a congenital or acquired primarily non-glomerular disorder of the kidneys, with chronic damage, many shrunken or globally sclerosed glomeruli in one, and many fetal glomeruli in the other, but no definite evidence of diabetic nephropathy. One child (case 14, reported previously [16]) had normal kidneys on ultrasound examination at ages 4 months and 7 years but had small kidneys at 10 years of age, the right on the fifth centile and the left on the 25th centile, with scarring of the upper pole of this kidney on a dimercaptosuccinic acid (DMSA) scan. No material was available for electron microscopy. The other child (case 11) had symmetrically small kidneys on a DMSA scan.

One child (case 9) had pauciimmune necrotizing crescentic glomerulonephritis associated with anti-neutrophil cytoplasmic antibodies (ANCAs) against myeloperoxidase, and presented with severe acute kidney injury, already with extensive chronic kidney damage. She remained on dialysis until kidney transplantation, 3 years after presentation.

The other child (case 16) had IgA nephropathy and was biopsied to investigate kidney impairment with proteinuria. There was already a moderate amount of chronic kidney damage. Kidney impairment progressed but proteinuria improved.

## Discussion

The 17 children in our series all had clinical indications for kidney biopsy. In contrast, there was no clinical kidney abnormality apart from microalbuminuria in eight patients among the 243 biopsied by the International Diabetic Nephropathy Study Group in what was almost certainly the largest series of kidney biopsies in children with type 1 diabetes, although the number of children was not specified in the series of patients aged 10 to 40 years at biopsy (mean 16.8 years) [17].

In that study, detailed morphometry on electron micrographs showed, among other things, that the prevalence of thickening of the glomerular basement membrane and of enlarged mesangial volume both increased in proportion to the duration of type 1 diabetes [18]. The morphometric methods used in that study were unrealistic in everyday practice and were to detect the earliest changes of diabetic nephropathy before these were clinically evident [11]. Accordingly, the assessment of diabetic nephropathy that we used, specifically, judgment of glomerular basement membrane thickness, was practical but crude and subjective [13], and we may have missed early and subtle changes of this in association with recognisable glomerular disorders. Even in the Renal Pathology Society classification of diabetic nephropathy [7], how the thickness of glomerular basement membranes should be measured was not standardised, and so the identification of the earliest stages of thickening of basement membranes may be difficult in routine practice because the definition is to some extent arbitrary and accurate measurement is not easy.

Only two other series appear to have been published reporting findings in type 1 diabetic children who had a kidney biopsy for a clinical abnormality, both investigating the nephrotic syndrome, and both with few biopsies: two [19] and five [20]. Diabetic nephropathy has been reported in children with type 1 diabetes even before clinical abnormalities ascribable to diabetes mellitus were apparent [18], although this does not mean that clinical abnormalities will necessarily develop. In another study that used a sophisticated morphometric method [11], in 71 initially normoalbuminuric type 1 diabetic patients, mostly adults, after a mean of 19.1 years of diabetes at kidney biopsy and after a mean of 12.7 years of follow-up, two thirds of those with thickened basement membranes remained normoalbuminuric, while only one third progressed to develop proteinuria or kidney failure [21].

An electron microscopic feature that we found has been overlooked in most accounts of diabetic nephropathy, but was described in 1964 by Dachs et al. [22]. This was a change they called “membranous transformation,” in which the epithelial aspect and, to a lesser degree, the endothelial aspect of thick glomerular basement membranes were irregularly scalloped or studded with spike-like projections, as in our Fig. 6, and as illustrated in human diabetic nephropathy [23] and experimental diabetes mellitus in rats [24]. The irregularity of the projections, their scattered distribution on basement membranes, and the lack of immune deposits on immunohistological study differentiated this change from membranous nephropathy.

Four children in our series had diabetic nephropathy on its own, and three had diabetic nephropathy and IgA nephropathy. This combination has been reported in adults [3, 25, 26], and there has been speculation that IgA nephropathy is commoner in diabetic patients than in non-diabetic people [27]. We also had one child apparently with IgA nephropathy on its own. This was an example of the well-known finding of non-diabetic kidney diseases in diabetic patients, much more commonly reported in adults than in children [3, 19, 20, 25–29]. In diabetic children, minimal change disease has been one of the commonest conditions reported [19]. Clinical clues in our series that a kidney biopsy may show something other than diabetic nephropathy included acute kidney injury, either microscopic haematuria or chronic kidney impairment with little or no proteinuria, and the nephrotic syndrome with a relatively short duration of diabetes (Table 1).

Two conditions worth special mention are (1) membranous nephropathy occurring with IgG4 antibodies to CASPR2 and LGI1, which are associated with neurological problems although an association with membranous nephropathy does not appear to have been reported previously [14], and (2) Wolcott–Rallison syndrome. In this syndrome, the most usual way in which the kidneys are affected is by episodes of acute, reversible kidney dysfunction when there is liver dysfunction [15]. Permanent structural changes in the kidney have only rarely been reported [15, 30], and in particular, diabetic nephropathy seems not to have been reported.

We have shown that (1) overt diabetic nephropathy either on its own or combined with IgA nephropathy can be found in children, but is rare, and (2) kidney disorders other than diabetic nephropathy can be found in children with type 1 diabetes mellitus.

### Supplementary Information

Below is the link to the electronic supplementary material.

Graphical abstract (PPTX 45 KB)

## Data Availability

All relevant information is included in the paper.
