# Supplementary material for: Kidney biopsy findings in children with diabetes mellitus
Source: Pediatr Nephrol. 2023 Dec 21;39(6):1865–73. doi: 10.1007/s00467-023-06254-9 (PMC11026184; doi:10.1007/s00467-023-06254-9)
Supplement: Supplementary file 1 — Graphical abstract (PPTX 45 KB) [file 467_2023_6254_MOESM1_ESM.pptx]

## Slide 1
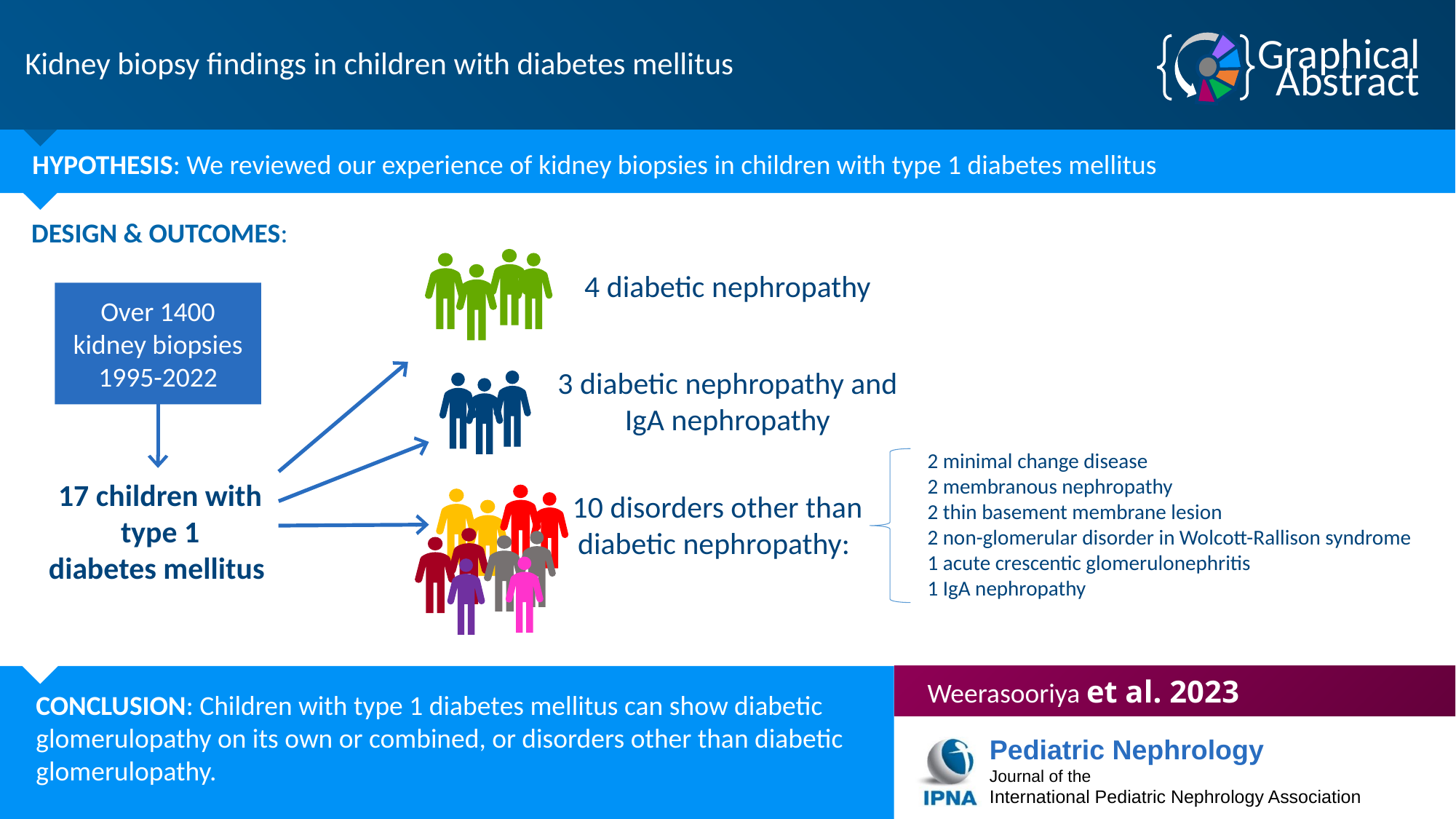

Kidney biopsy findings in children with diabetes mellitus
HYPOTHESIS: We reviewed our experience of kidney biopsies in children with type 1 diabetes mellitus
DESIGN & OUTCOMES:
4 diabetic nephropathy
Over 1400 kidney biopsies
1995-2022
3 diabetic nephropathy and IgA nephropathy
2 minimal change disease
2 membranous nephropathy
2 thin basement membrane lesion
2 non-glomerular disorder in Wolcott-Rallison syndrome
1 acute crescentic glomerulonephritis
1 IgA nephropathy
17 children with type 1
diabetes mellitus
10 disorders other than diabetic nephropathy:
Weerasooriya et al. 2023
CONCLUSION: Children with type 1 diabetes mellitus can show diabetic glomerulopathy on its own or combined, or disorders other than diabetic glomerulopathy.
